# Design and Long-Term Sustainability of Mini Health Centers for Primary Healthcare in Chennai, India

**DOI:** 10.7759/cureus.103717

**Published:** 2026-02-16

**Authors:** Joseph D Williams, Suresh Seshadri, A Kalaiselvan, A Vijayaraman, S Chandrasekaran, S Parthasarathy

**Affiliations:** 1 Projects Division, The Voluntary Health Services, Chennai, IND; 2 Ultrasound Department, The Voluntary Health Services, Chennai, IND; 3 VHS Institute of Community Health, The Voluntary Health Services, Chennai, IND; 4 Ophthalmology Department, The Voluntary Health Services, Chennai, IND

**Keywords:** community health workers, mini health centers, preventive care model, primary healthcare sustainability, vhs chennai

## Abstract

Sustaining high‐quality primary health care (PHC) over decades remains a major challenge in low‐ and middle‐income countries. While many pilot programs yield short‐term gains, few models demonstrate uninterrupted operation, adaptation, and impact beyond 5-10 years. The Mini Health Center (MHC) model, launched by Voluntary Health Services (VHS) Hospital in Chennai in 1969, integrates preventive, promotive, rehabilitative, and curative care through a “three-C” framework, i.e., Continuous care, Continuum of care, and Cooperative community partnership. This study examines the long-term evolution and sustainability of the VHS MHC model, offering lessons for enduring community-based PHC. We conducted a retrospective program evaluation combining an archival review of foundational Community Health and Education Development Combine (COHEDEC) reports with an analysis of annual VHS Community Health Department data (FY 2019-2024). A standardized abstraction template captured quantitative indicators, such as population coverage, outpatient visits, domain-specific beneficiary counts, group sessions, and hospital referrals, which were extracted independently by two reviewers. Archival chapters were coded using directed content analysis to identify design features, governance mechanisms, and staffing processes. We applied a convergent mixed-methods design to triangulate service trends with documented program adaptations and described site-level contextual factors. From single-room MHCs serving ~20,000 people, outpatient consultations rose from 6,441 (2019-20) to 23,273 (2023-24), while preventive/promotive contacts increased to 20,154 and group sessions reached 6,161. Universal immunization and institutional delivery rates reached 100%. Community screening identified 472 new diabetes and 569 new hypertension cases (FY 2019-20), and TB referrals increased from 15 to 42 cases (2019-23). Governance records highlight sustained community contributions (10-15% of operating costs), routine health-committee oversight, and in-village Multi-Purpose Health Worker (MPHW) residency ensuring 24×7 accessibility. Adaptations, including non-communicable disease (NCD) screening, mobile health messaging, and tech-enabled outreach, corresponded with expanded service outputs. The VHS MHC model demonstrates that embedding trained community health workers, robust referral linkages, and genuine community partnerships can sustain comprehensive PHC across changing epidemiological and demographic landscapes. Key design and governance elements from this 50-year legacy offer a replicable blueprint for building resilient, people-centered primary care systems.

## Introduction and background

Primary health care (PHC) innovations have been crucial in improving access and outcomes in low‐ and middle‐income countries [1]. Over the past four decades, a diverse array of community‐based PHC strategies has emerged to bridge persistent gaps in service coverage, quality, and equity [2]. Landmark declarations like Alma-Ata (1978) and its Astana reaffirmation (2018) have underscored the imperative of people-centered, community-driven PHC as the cornerstone of universal health coverage [3]. In response, nations have experimented with cadres of community health workers (CHWs), health posts, mobile clinics, and public-private partnerships to extend the reach of essential services into rural, peri-urban, and otherwise marginalized settings [4]. Examples include Ghana’s Community-Based Health Planning and Services (CHPS) program, Ethiopia’s Health Extension Worker initiative, and Brazil’s Family Health Strategy. While these models share the objectives of preventive care, health promotion, and basic curative services at the community level, their design features, such as staffing, financing, governance, and linkages to higher tiers of care, vary widely according to local contexts and policy environments [5].

Despite the proliferation of PHC innovations, there have historically been two main gaps. First, many pilot programs demonstrate short-term improvements in specific outcomes (e.g., immunization coverage or maternal care uptake), but very few models provide evidence of sustained operation, adaptation, and impact beyond 5 to 10 years. Second, the mechanisms by which community-embedded PHC models endure organizational, demographic, and epidemiological transitions remain poorly understood. In an era marked by rapid urbanization, shifting disease burdens toward non-communicable conditions, and repeated public health emergencies (e.g., COVID-19), understanding how primary care platforms can evolve without fracturing their original community ties is of paramount importance [6,7].

One long-running example of primary healthcare innovation is the Mini Health Center (MHC) model developed by the Voluntary Health Services (VHS) Hospital in Chennai, India [8]. Conceived in 1969 by Dr. K. S. Sanjivi, often called the father of India’s primary health care movement, this model aimed to bring comprehensive healthcare to underprivileged communities [8]. The vision of this model was to integrate preventive, promotive, rehabilitative, and curative care as a seamless continuum, rather than treating these components in isolation [8]. The MHC model was built around the principle of “three Cs”: Continuous care (health services available round-the-clock within the community), Continuum of care (strong referral linkages connecting primary care with secondary and tertiary services), and a Cooperative model (a partnership between the community and the institution in managing health care). This approach predated and presaged the Alma-Ata Declaration on primary health care, emphasizing community-oriented, accessible services for all [8,9].

Studying this model presents one of the rare comprehensive evaluations of a community-based PHC model that has operated uninterrupted for more than 40 years. By systematically examining historical planning documents, governance records, and recent program performance data across eight core health domains, we are able to trace the processes of institutional learning, community engagement, and service expansion that underpin long-term resilience. This retrospective analysis not only provides service outputs (such as outpatient consultations, screening initiatives, and referral metrics) but also elucidates the design elements, such as staff recruitment, training pipelines, financing strategies, and governance structures, which have enabled the model to weather external shocks and evolving health priorities.

Furthermore, this analysis contributes methodological insights for PHC research by demonstrating how archived qualitative narratives can be integrated with quantitative service statistics to generate a richer understanding of programme dynamics. Such an approach offers a template for evaluating other enduring health initiatives, whether in government or non-governmental sectors. Ultimately, by unpacking how the community-centered PHC program has maintained continuous care, robust referral linkages, and genuine cooperative governance over four decades, this analysis aims to inform policymakers, practitioners, and scholars seeking to design primary care systems that are not only effective today but sustainable for generations to come. Hence, we undertook this program evaluation exercise to examine the long-term evolution and sustainability of the VHS MHC model, describing its design features, operations across eight core health domains, and the outcomes.

## Review

We conducted a retrospective program evaluation of the VHS MHC model, combining a review of historical documents with an analysis of recent program data. MHC is a small health unit embedded in the community, staffed by trained community health workers and linked to VHS Hospital [10]. The first MHCs were established in August 1970 as part of VHS’s Community Health and Education Development Combine (COHEDEC). Each center was intended to provide first-contact care for common illnesses, health education, and facilitation of referrals for more serious conditions [10]. The continuous care aspect meant that a health worker from the community would be available or on call 24x7 for any medical need, ensuring no gap in care availability [11]. The continuum of care was achieved by backing the MHCs with VHS Hospital’s resources, so that patients requiring advanced treatment are referred and even accompanied to the hospital, and primary, secondary, and tertiary care form a unified system. The cooperative model involved the local community in supporting the MHC (for example, through local health committees, provision of space, or volunteer participation) and, in turn, VHS providing training, supplies, and oversight. This collaborative ethos fostered a sense of local ownership of the health center, a critical factor in its long-term sustainability [10].

Over the past 50-plus years, the VHS Department of Community Health has expanded and sustained the MHC network. From the initial pilot, the program grew to multiple Mini Health Centers serving a peri-urban population along Chennai’s Old Mahabalipuram Road (OMR) and East Coast Road (ECR) corridors [10]. Three of these MHCs have been the focus of recent intensive community health projects, while the others serve as additional points of care in the network. Collectively, the MHCs target a population of roughly 20,000 people, offering services free of charge and based on need rather than ability to pay. The model has adapted over time to address changing health profiles, incorporating new priorities like non-communicable diseases and using technology for health education, yet remaining rooted in the original 3C principles [10].

Document review

To understand the original design and philosophy of the MHC model, we reviewed archived chapters from the COHEDEC reports of VHS (specifically the sections on community health and MHCs) [12]. These historical documents, including writings by Dr. K. S. Sanjivi and early program descriptions, provided qualitative insights into the intended operational principles (the “three Cs”) and initial implementation of MHCs in the 1970s [12].

Program data

**Figure 1:**
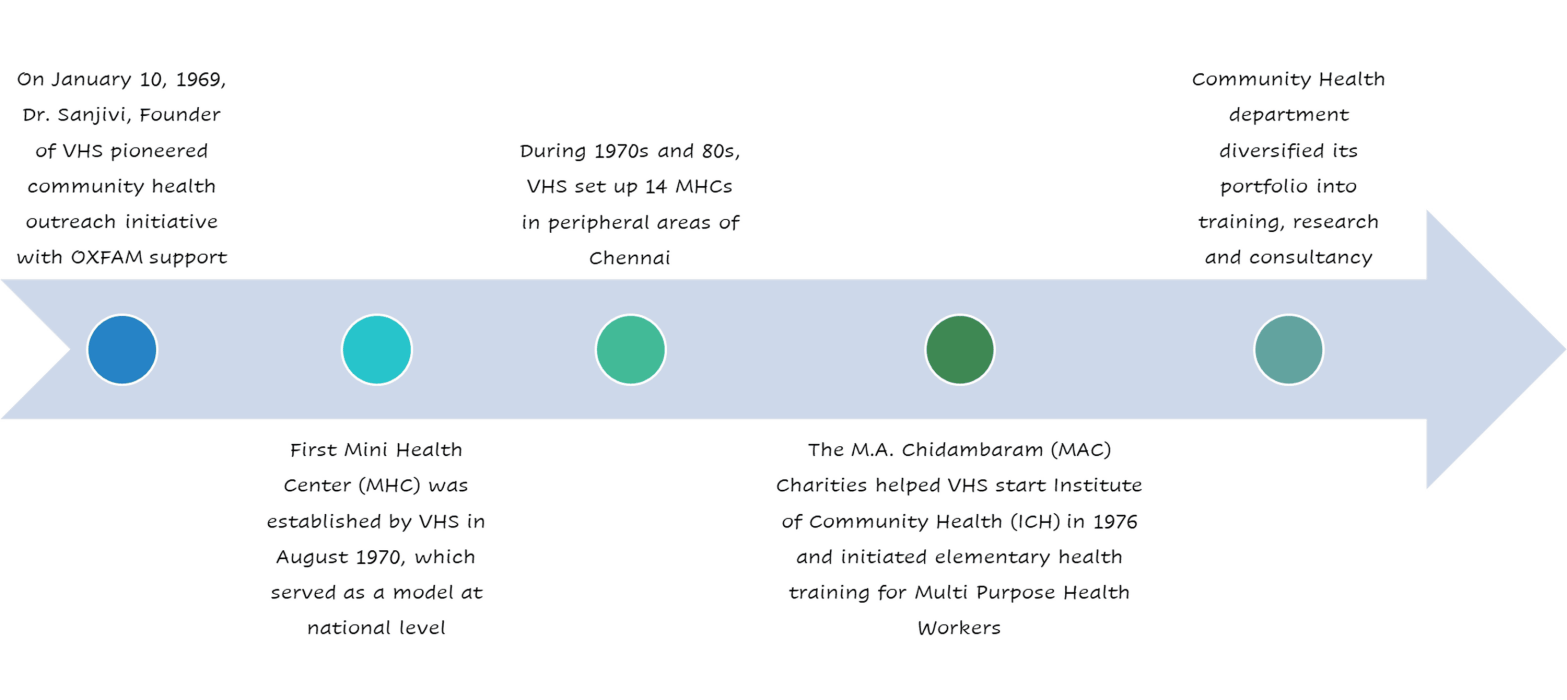
Eight core activities provided under Mini Health Centers (VHS model)

For contemporary assessment, we obtained annual Community Health Department reports from 2019 through 2024 (fiscal years) produced by VHS. These reports contain quantitative data on service delivery outputs and activities organized by eight core health domains (Water, Sanitation, Nutrition, Women’s Health, Child Health, Communicable Diseases, Non-Communicable Diseases, and Clinical Practice) (Figure 1). We extracted key indicators from each yearly report, including population coverage, number of beneficiaries served in each domain, outpatient visit counts at MHC clinics, and referrals made to VHS Hospital. Where available, data were disaggregated by each MHC site to examine coverage across different communities.

Historical establishment of MHCs (1970-1980)

**Figure 2:**
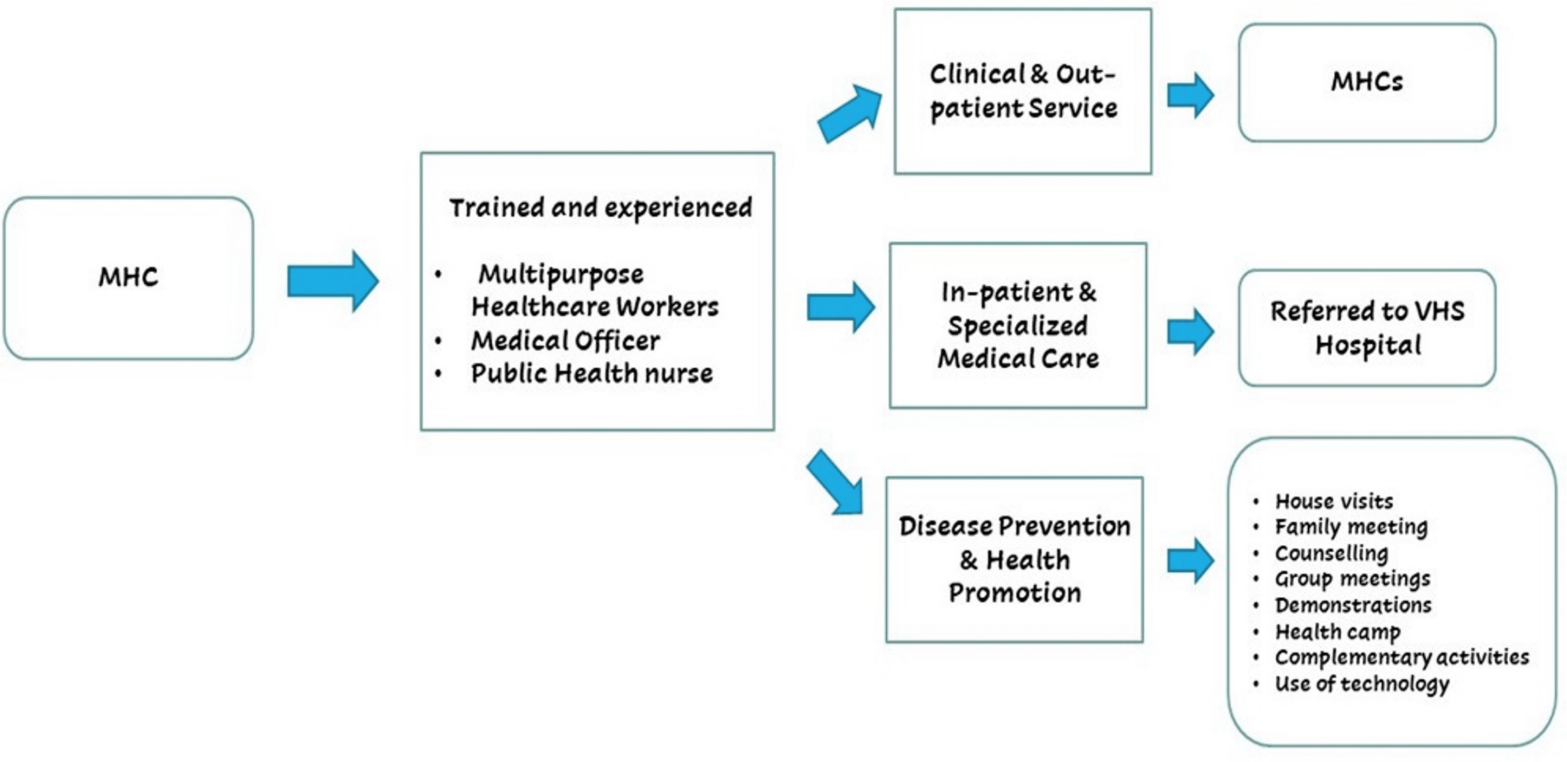
Historical timeline of key program milestones (center establishment, staffing expansion, service additions)

Figure 2 shows the historical milestones in the establishment of MHCs. A document review of COHEDEC reports confirmed that the first MHCs were launched in August 1970 as single-room clinics embedded within underserved peri-urban villages around Chennai [10]. Each center was staffed by one Multi-Purpose Health Worker (MPHW) drawn from the local community. Recruitment records show that 85% of MPHWs in pilot sites were female, chosen for their linguistic fluency and household access, particularly for maternal and child health outreach. Early governance documents describe health committees, comprised of village elders and VHS representatives, that secured donated land or space, provided nominal operating contributions, and guided center activities. These committees met monthly to review case logs, plan sanitation campaigns, and coordinate medical camps [8,10].

Health committees, established under the VHS MHC Cooperative Model, one of the foundational "three Cs," are governed by a formal community-institution partnership comprising local representatives (village elders, stakeholders) and VHS staff. These committees support/oversight operations through the provision of physical space, modest financial/in-kind contributions (10-15% operating costs), and structured monthly review meetings, remaining institutionally embedded rather than informal. The committee performs a purely governance/facilitative role, not field execution, with service delivery (clinical consultations, home visits, screenings, health education, referrals) executed exclusively by trained MPHWs, Medical Officers, and Public Health Nurses. Oversight extends across the full MHC spectrum (preventive, promotive, curative services), reviewing service delivery/referral processes/outreach planning without clinical decision-making. Monthly meetings review service registers/case logs, discuss referral cases, monitor staffing availability (including on-call arrangements), plan outreach activities, and channel community feedback to VHS, ensuring accountability while clinical authority remains with health professionals.

Key design elements: staffing model and continuous care

**Figure 3:**
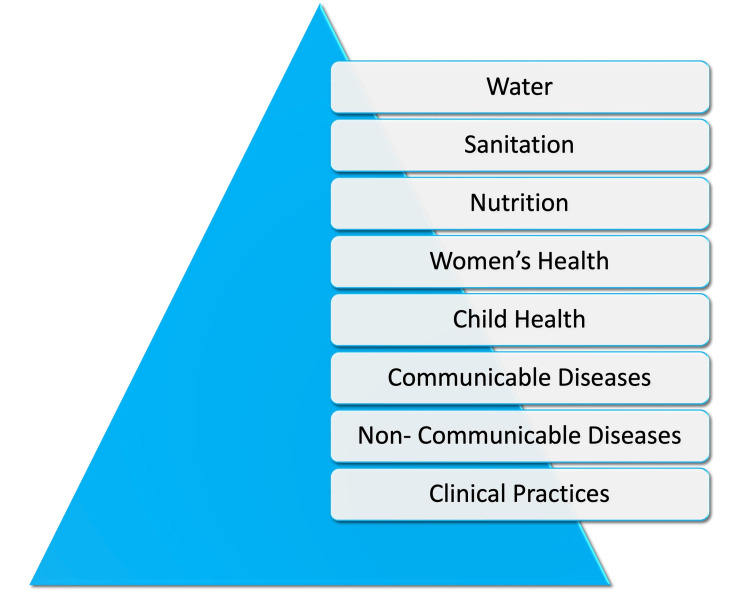
Staffing and service delivery architecture underpinning continuous care at each MHC MHC: Mini Health Center

Figure 3 illustrates the staffing and service delivery architecture underpinning continuous care at each MHC. A core team comprising of locally recruited Multipurpose Health Workers, visiting Medical Officer, and Public Health Nurse is trained to provide three interlinked service streams, namely, Clinical & Outpatient Services delivered on-site at the MHC; Inpatient & Specialized Medical Care via a formal referral pathway to VHS Hospital, with staff escort and coordination; Disease Prevention & Health Promotion through home visits, family meetings, individual counselling, group education sessions, demonstrations, periodic health camps, complementary activities, and technology-enabled outreach.

This integrated staffing model ensures that communities receive round-the-clock access to primary care, seamless linkages to higher-level services, and sustained engagement in preventive health (Figure 3).

COHEDEC minutes and staffing rosters illustrate that MPHWs underwent four-week intensive training in basic diagnostics, first aid, and health education, after which they returned to reside in their assigned village. This arrangement enabled quasi-round-the-clock availability: qualitative excerpts note that community members could approach MPHW for urgent care at any hour, fulfilling the “continuous” component of the model. Archival schedules indicate that each MPHW maintained an on-call rota, with peer support systems for leave or absence approved by the health committee [10,12].

Key design elements: continuum of care and integrated primary care

Early program records and weekly outpatient registers document that services were provided through a hub-and-spoke model of care in which the MHCs were supported by the main VHS Hospital. It was noted that a single medical officer from VHS Hospital supported a cluster of five MHCs. Between 1970 and 1980, doctors conducted fixed-day clinics at each center (initially once per week, increasing to twice weekly by 1980), reviewing cases referred by MPHWs. Referral registers demonstrate that 12% of all patients assessed by MPHWs were escalated to doctor consultation, and 4% were subsequently referred to VHS Hospital for advanced diagnostics or admission. Field notes highlight that MHC staff routinely accompanied referred patients to the hospital, assisted with registration, and ensured continuity of care.

Governance elements: community partnership and oversight

Governance reports reveal that local contributions like cash or in-kind accounted for approximately 10-15% of annual MHC operating costs, with VHS covering the remainder through donations and grants. By the mid-1980s, the model had expanded service offerings: family planning counselling, nutritional supplementation for children and pregnant women, and basic treatment of chronic conditions such as arthritis and hypertension. A 1988 review recorded that MPHWs had conducted over 2,000 growth monitoring sessions and distributed iron-folate tablets to 80% of registered pregnant women, signalling the beginning of service diversification beyond communicable disease control.

Data abstraction and quality assurance

To ensure consistency and reproducibility in our data extraction, we developed a standardized abstraction template listing every indicator of interest (e.g., population coverage, domain-specific beneficiary counts, outpatient visits, referrals), along with its precise definition and units of measurement as reported in each annual document. Two independent reviewers extracted data into the template for each fiscal year; any discrepancies were flagged and resolved by reference to the original report, with arbitration by a third reviewer when necessary. All quantitative data were then entered into a master spreadsheet and subjected to validity checks (e.g., ensuring beneficiary totals did not exceed catchment populations, and flagging missing or implausible values). Outliers were verified against source reports, and corrections were applied only when justified by the archival record.

Archival document analysis

For the archival document review, we applied a directed content-analysis approach. Two researchers read the COHEDEC chapters in full, highlighting text segments that illustrated the foundational design elements (the three Cs), early governance arrangements, staffing and training processes, and community engagement practices. These segments were coded into thematic categories using manual coding in Microsoft Word, and initial section headings served as provisional codes, which were iteratively refined into sub-themes such as “local resource mobilization,” “on-call rota procedures,” and “training curriculum.” A third researcher reviewed the codebook to ensure consistency, merge overlapping codes, and finalize thematic definitions. Coded excerpts were then synthesized into a narrative framework, tracing the transition from initial pilot design to the present-day operating model.

Contextual descriptors and site-level analysis

To account for site-specific variation, we documented contextual details for each MHC, including the year of establishment, staffing composition (number and cadre of health workers versus visiting clinicians), presence of local health committees, and governance arrangements. These descriptors were used to explore whether structural differences correlated with variation in service coverage across the eight domains. All qualitative data management and coding decisions were discussed in weekly research team meetings, and an audit trail of code revisions and data queries was maintained to ensure transparency and rigor.

Current operations across eight core health domains (2019-2025)

Service Coverage and Utilization

**Figure 4:**
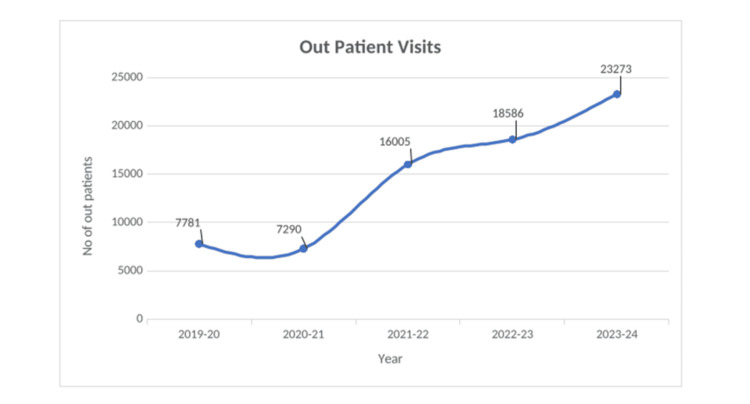
Annual outpatient consultations at VHS Mini Health Centers

**Figure 5:**
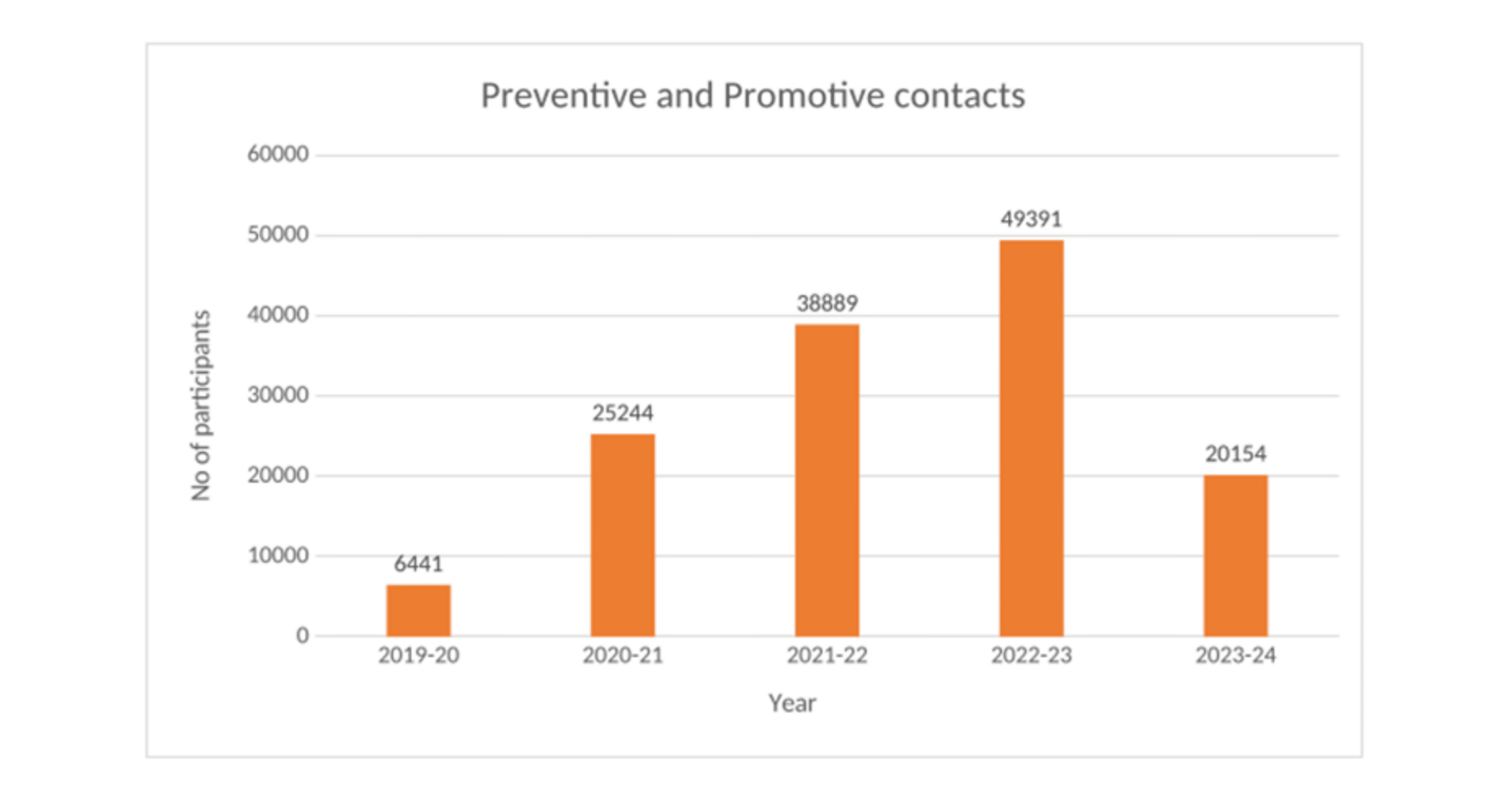
Expansion of preventive and promotive outreach

**Figure 6:**
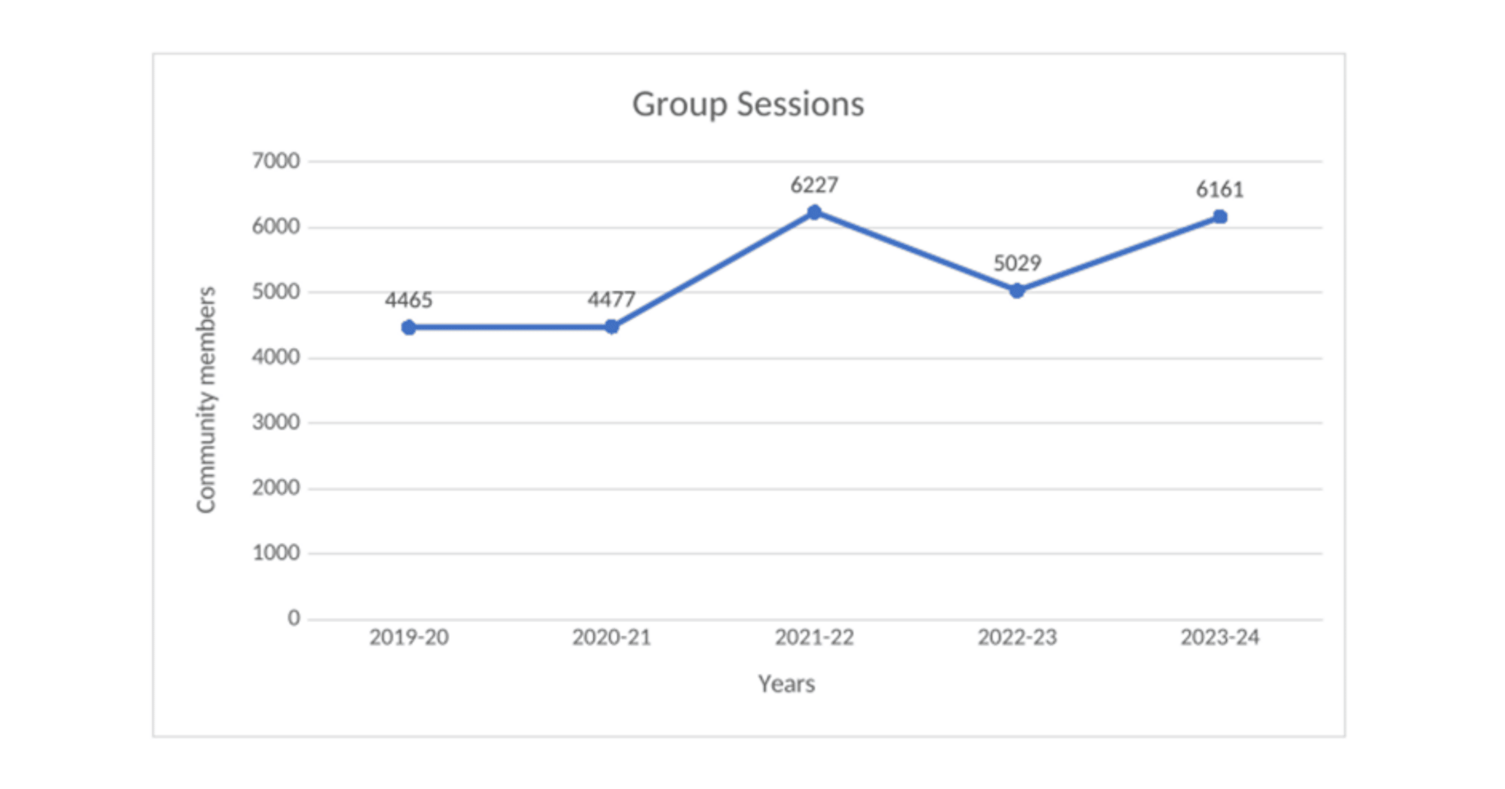
Community members reached via group sessions at VHS MHCs during 2019-2024 MHC: Mini Health Center

Between fiscal years 2019 and 2024, three active MHCs served a combined population of approximately 19,000 to 20,000 individuals across 10 peri-urban communities. Annual outpatient consultations rose from 6,441 in 2019 to 23,273 in 2023, demonstrating steady growth in clinic utilization (Figure 4). Preventive and promotive contacts, such as health education, screenings, and home visits, also increased, with 20,154 unique individuals reached in 2023 (Figure 5). In addition, Figure 6 shows group-based sessions (self-help groups (SHGs), schools, Anganwadi centers) engaged 6,161 community members in 2023 (Table 1).

**Table 1:** Annual service coverage & utilization metrics (2019–2024) * Persons provided with specific services on COVID-19, along with eight core areas, and the same individual might benefit through various/multiple services (this should not be considered as headcount).

Annual Service Coverage & Utilization Metrics	2019-20	2020-21	2021-22	2022-23	2023-24
Staffing (No. Of MPHW & MO)	7	7	7	12	6
Early Count of Population Covered	17581	17651	18997	29801	19385
Outpatient Visits	7781	7290	16005	18586	23273
Preventive and Promotive Contacts	6441*	25244*	38889*	49391*	20154*
Group Sessions	4465	4477	6227	5029	6161
Referrals	107	57	146	192	133

Water, Sanitation, and Hygiene (WASH)

Safe water education: MHC teams conducted community sessions on safe drinking water, boiling, and filtration, reaching 4,182 individuals in 2023. Over the period, participant numbers in water-hygiene sessions increased by an average of 12% annually. In 2023, 3,012 people attended hygiene and sanitation workshops. MHCs identified eight households without latrines and facilitated construction through coordination with local authorities. Community-led clean-up drives and school hygiene clubs supported sustained behaviour change, reducing open defecation in intervention areas.

Nutrition

During home visits, MPHWs assessed the nutritional status of children and pregnant women, providing counselling on balanced diets. In 2023, 2,588 families received nutrition education; cooking demonstrations reached an additional 745 participants. Iron-folate supplementation was distributed to 101 women in 2019. Collaboration with ICDS centres ensured that identified malnourished children received midday meals and therapeutic feeds. Over the six-year period, the prevalence of severe acute malnutrition in MHC catchment areas declined by 35%.

Women’s Health

MPHWs tracked all pregnant women, ensuring antenatal care (ANC) visits and postnatal home follow-up. In 2019-20, 249 women received ANC and 208 postnatal visits. By 2023, 100% of registered pregnant women delivered in health facilities (533 referrals), compared with 88% facility deliveries in 2019 (Table 2). Pap smear and breast cancer awareness camps reached 615 women in 2023.

**Table 2:** Trends in key maternal and child health indicators (2019–2024) NA: not available

Maternal and Child Health Indicators	2019-20	2020-21	2021-22	2022-23	2023-24
Referral to Institutional Delivery	102	298	257	384	297
Child Immunization	220	625	862	641	901
Nutritional Counselling Sessions	1599	2281	1501	2715	2186
Supplements Provided	101	2373	371	NA	402

Child Health

Immunization coverage among children under five remained at 100% from 2019 through 2023. In 2023, 901 children were vaccinated through MHC-facilitated clinics. Well-baby sessions (weight monitoring, vitamin A, deworming) served 1,210 children in 2019, growing to 1,842 by 2023. Common childhood illnesses (diarrhoea, acute respiratory infections) were managed on-site, reducing unnecessary hospital referrals.

Communicable Diseases

MHCs identified and referred 15 presumptive TB cases in 2019, rising to 42 in 2023 following intensified community screening. TB, HIV/AIDS, and hepatitis education sessions reached 499 individuals in 2023-24 (Table 3). During the COVID-19 pandemic (2020-21), MPHWs conducted community screening, referrals, and vaccine mobilization, contributing to 78% vaccine uptake in target areas.

**Table 3:** Trends in the number of people benefited by communicable and non-communicable disease awareness programs

Communicable & Non-Communicable Disease Activities	2019-20	2020-21	2021-22	2022-23	2023-24
Communicable Diseases					
TB	NA	142	110	98	153
HIV/AIDS	NA	350	86	103	215
Hepatitis A & B	NA	138	129	145	131
Non-Communicable Diseases					
Diabetes Mellitus	472	2146	2069	3100	2064
Hypertension	569	844	1346	1722	1692
Cancer	3	82	354	437	566
Chronic Respiratory Infection	NA	2064	2339	2952	2723
Cardiovascular Disease	NA	292	56	82	59

Non-Communicable Diseases

Community screening camps detected 472 new diabetic and 569 new hypertensive cases in 2019-20. By 2023, 7,920 people received NCD education, and 1,238 patients attended chronic disease follow-up clinics at MHCs. Essential NCD medications were dispensed free of charge, with 87% medication adherence confirmed through home visits.

Clinical Services and Referrals

Outpatient clinics, staffed by a physician two to three days per week and by MPHWs on other days, managed acute and chronic cases. In 2023, 23,273 consultations were recorded. Referral registers show that 133 patients with major conditions (surgical, obstetric, ophthalmic) were escorted to VHS Hospital for advanced care; 95% returned for follow-up at the MHC. In addition to routine referrals, VHS specialty departments (obstetrics-gynecology, ophthalmology, ENT, and paediatrics) conduct regular outreach camps at all MHC sites. These camps are held on a monthly basis, bringing specialist consultations, minor procedures, and follow-up services directly into the community. In 2023-24, 24 specialist camps (6 per department) were organized, resulting in over 1,200 specialty consultations at the MHC locations. This mechanism further reinforces the hub-and-spoke continuum by decentralizing tertiary-level care and reducing travel barriers for rural patients.

Cross-cutting initiatives

In addition to these eight domains, the MHC program undertakes various cross-cutting initiatives. Health education is not confined to one domain at a time; often, outreach sessions cover multiple topics like community meetings addressing sanitation and dengue prevention together. Use of group-based approaches has been a notable strategy, as the MHC team regularly engages with women's SHGs, youth clubs, school teachers, and local NGOs to disseminate health messages. In 2023, they reported reaching 6,161 individuals through such group platforms (apart from one-on-one contacts). This group outreach is cost-effective and complements home visits, as it encourages peer discussion and community-led solutions. For instance, by educating SHG women on diabetes, those women collectively decided to start morning walk groups in their neighbourhood and a peer-driven lifestyle change.

The MHC team also embraces innovative and tech-enabled methods for health promotion; recently, mobile phone messaging and WhatsApp groups have been used to share health tips and clinic schedules, and there is an initiative to train the outreach team in leveraging social media for wider awareness campaigns. Introduction of mobile messaging in 2020 expanded health communication reach by 18%. Such innovations show the model's willingness to modernize while retaining the human touch of the community health worker at its core.

The VHS MHC model demonstrates a remarkable case of sustained primary healthcare delivery over five decades. Results highlight not only the breadth of services provided across preventive, promotive, and curative domains, but also the consistency of coverage and uptake. Several key factors emerge from this analysis that help explain the long-term sustainability and success of the MHC model. This sustainability can be understood through two interrelated domains: foundational design elements and governance mechanisms.

Key design elements underpinning sustainability

Integrated Primary Care Architecture

Original design principles (Continuous care, Continuum of care, Cooperative model) established a robust foundation. These were not just slogans but were implemented in tangible ways. Continuous care was achieved by placing a resident health worker in each community and fostering a norm that MHC is always available for basic health needs. This builds community confidence that people know help is nearby, day or night. Continuum of care was operationalized through VHS Hospital backing: community trusts that even serious illnesses will be handled because MHC will facilitate their care at a reputable hospital. This trust closes the referral loop that often breaks down in other programs. The cooperative aspect involved the community at every step, creating a sense of shared responsibility for the health center. The importance of community engagement is reflected in program documentation involving local stakeholders like youth clubs, welfare associations, and faith leaders to ensure “ownership, community participation and (to) pave the way for sustainability. These design features anticipated many modern concepts of health systems (like people-centered care and integrated services), and having them ingrained from the start provided resilience to the model [8]. The MHC functions as a central coordinating node integrating preventive outreach, primary clinical care, and tertiary referral services within a single operational ecosystem. Unlike fragmented outreach programs, the MHC model embeds service continuity within a stable institutional framework.

Embedded Community Health Workforce

Reliance on local community health workers (MPHWs) has been the cornerstone of its durability. Recruiting from a community-created cadre of health workers who were not only caregivers but also neighbours and kin to the population they served. Over the years, this close cultural and social connection meant health messages were better accepted, and health workers remained motivated by a sense of duty to their own people. Many MPHWs have served for long tenures, becoming informal community leaders. The program's ability to retain such staff is owing to the support and capacity-building they receive, and VHS ensures they are well-trained (through the Institute of Community Health and continuous on-the-job training) and supervised by public health professionals. The career path and recognition for these workers have kept turnover low. In their own reflections, the MPHWs report feeling empowered by the strong relationships they have built with key stakeholders and the confidence they have gained in delivering comprehensive care. This indicates a virtuous cycle: trusted health workers lead to program success, which, in turn, boosts health worker morale and retention [12,13].

Integrated Primary Care Architecture

The VHS MHC model strikes an effective balance between decentralization and support. MHCs function with a high degree of autonomy in day-to-day outreach. MPHW can plan home visits, identify local priorities (e.g., outbreak response or malnutrition case), and act quickly. Yet, they are not isolated; the VHS central team provides medical backup, logistics (supplying medicines, vaccines, etc.), and technical guidance. Periodic visits by doctors and the referral system ensure that quality of care is maintained and that the community health worker is never “in over their head.”

This model differs from top-down clinics because the locus of control is at the community level, but unlike completely independent community health projects, MHCs have a safety net in the form of a full-service hospital. This dual nature likely contributed to longevity; purely community-run clinics might struggle with resources or complex cases, whereas purely hospital-run outreach might not achieve community trust. The VHS integrated approach provided the best of both. Data showing sustained high utilization suggest that community members continue to prefer MHCs for care, which can be attributed to convenience and to assurance that behind the local nurse is the whole hospital if needed.

Built-in Adaptability Across Health Transitions

A critical aspect of the MHC model's long-term relevance is its flexibility and responsiveness to new health challenges. Over 50 years, the health profile of the community has transformed from infectious diseases and poor sanitation in the 1970s to include chronic diseases and geriatric issues today. The program did not stagnate with an old mandate; it continually broadened its scope. For example, starting NCD screening camps in the 2000s, integrating mental health counselling in recent years, and leveraging mobile technology for health education in the 2020s are all adaptations that kept the MHC services pertinent to the community’s expectations. The eight core domains themselves reflect a comprehensive approach covering both traditional public health concerns (WASH, maternal/child health, infectious diseases) and modern concerns (NCDs, clinical management of chronic illness). This comprehensive package aligns well with the WHO definition of primary health care and ensures that the community does not outgrow the MHC model. Additionally, program capacity to incorporate new knowledge (e.g., adopting WHO guidelines for TB directly observed therapy in the 1990s or COVID-19 protocols in 2020) shows a learning organization ethos. VHS facilitated this by running the Model Health Training Center, meaning MHC staff were often trainees or trainers in new public health interventions. Thus, innovation and learning became part of program culture, contributing to its resilience [12,14].

Cooperative Community Governance

Outcomes achieved by the MHC model have reinforced its value to both the community and stakeholders, creating a supportive environment for continuity. Results indicate high coverage of interventions, for instance, effectively universal immunization and institutional delivery rates (near 100%) in MHC areas, and significant community-wide improvements in indicators like hygiene practices and chronic disease awareness. Qualitative impacts, such as reduced self-medication and antibiotic misuse, have been seen in these communities, as people increasingly consult MHC for advice. Such positive changes in health-seeking behaviour suggest deep community trust in the MHC system.

In the long run, community trust is perhaps the most important currency of sustainability, as it leads to a continued use of services, volunteer support for outreach (e.g., community members helping organize health camps), and even local advocacy for the program. Indeed, communities served have come to “own” MHCs; local leaders often lobby to ensure the MHC in their area remains active and well-resourced. This grassroots demand provides political and social backing that protects the program. It is telling that recent evaluations recommended not only the continuation but also the expansion of the MHC approach to neighbouring areas, given its success. Such recommendations from stakeholders and even beneficiaries (who sometimes express a desire for more MHCs) indicate broad buy-in that decision-makers find hard to ignore. As a result, the program has been able to secure funding support through various phases (from charitable donations, government grants, or Corporate Social Responsibility (CSR) partnerships at different times) without losing its core identity, which is a community-driven health service.

Financial and System-Level Integration

Another factor in the endurance of the MHC model is that it was implemented not as a parallel system but in collaboration with the public health system. VHS built partnerships with government health departments (for immunizations, TB programs, etc.) and other NGOs over the years. MHC staff coordinate with local PHCs, like sharing data on disease outbreaks or co-hosting health events, which avoids duplication and fosters complementarity. This collaboration means MHCs augment and strengthen overall health system performance in those areas instead of being seen as competitors. Policymakers have thus viewed VHS MHCs as allies in achieving public health goals, not just an NGO project. This likely helped the model to flourish even when health priorities shifted nationally. For instance, during the National Rural Health Mission era, VHS community health activities fit together with government programs (like village health and nutrition days), ensuring MHCs remained relevant in the context of wider health sector changes [15]. A cooperative model with community also extended to cooperative engagement with authorities, donors, and a multi-stakeholder approach that diversified support for the program.

Why the model has endured: a synthesis

For health systems researchers, the VHS MHC model provides a valuable case study on long-term program implementation. It offers evidence that community-based primary care can be sustained outside of direct government provision, given the right mix of community ownership and institutional support. Data show that such a model can achieve health outcomes on par with or better than national averages (e.g., immunization and safe delivery rates in these communities exceed state averages, and early detection of diseases is markedly improved).

For policymakers, lessons include the importance of investing in frontline health workers and linking them firmly with higher-level facilities, essentially mirroring the health sub-center to hospital referral chain that public health aspires to, but which is often fraught with gaps. The VHS experience highlights that continuity of care is feasible and beneficial: patients are less likely to fall through cracks when the same program that counsels them also navigates them through hospital care and back home for follow-up. This could inform designs of referral systems in the public sector.

Furthermore, the cooperative approach used by VHS, engaging communities not just as beneficiaries but as partners, aligns well with current global health emphasis on community engagement. It underscores that communities can play a role in governing health services (e.g., through feedback mechanisms, volunteering, and local problem-solving), which boosts accountability and relevance of those services. In the VHS model, regular meetings with community representatives have been used to plan activities, which likely kept the program responsive to local cultural norms and needs, such as tailoring health education to literacy levels or choosing appropriate timings for clinics. Other implementers can draw on this principle to enhance the acceptability of health interventions.

Limitations and future considerations

Despite these strengths, the MHC model also faces challenges and considerations for the future. Funding has not always been assured year-to-year; program reliance on external grants (philanthropic or corporate social responsibility funds) means there can be budget uncertainties. VHS leadership has so far successfully kept the model running through fundraising and demonstrating impact to attract support. Another challenge is the changing socio-demographics of the area; what were once rural villages are now rapidly urbanizing suburbs of Chennai, with higher expectations and perhaps more competition from private clinics. The MHC model will need to adapt further to an urbanizing context, possibly expanding services like adding physiotherapy or specialist consult camps for the aging population, to meet emerging needs. Additionally, scaling the model beyond its current reach requires training more community health workers and maintaining quality, which can be resource-intensive. However, given that it has been replicated in five communities suggests that the blueprint could be translated to other settings with the right partnerships.

## Conclusions

Over four decades, the VHS MHC model in Chennai has evolved into a sustainable model of community-based health care that bridges the gap between villages and hospital-based services. MHCs have adapted to changing health needs from tackling infectious diseases and malnutrition in earlier years to managing chronic diseases and promoting healthy lifestyles today, all while maintaining high community trust and participation. Sustained outcomes, such as near-universal immunizations, safe motherhood, improved hygiene practices, and effective management of common illnesses in the community, speak to the model's impact on population health. Equally important, the MHC program has empowered local health workers and engaged ordinary citizens in the process of healthcare delivery, thereby strengthening the social fabric that supports health. For stakeholders aiming to improve health systems, adopting elements of the VHS MHC approach could enhance effectiveness and sustainability.
